# Case Report: A case of cutaneous polyarteritis nodosa in a child following a streptococcal infection

**DOI:** 10.3389/fped.2025.1553118

**Published:** 2025-03-13

**Authors:** Wei Cheng, Wei Yin, Fan Liu, Wen Yin

**Affiliations:** ^1^ Tongji Medical College, Huazhong University of Science and Technology, Wuhan, China; ^2^ Department of Rheumatology and Immunology, Wuhan Children’s Hospital Affiliated to Tongji Medical College, Huazhong University of Science and Technology, Wuhan, China

**Keywords:** children, group A β-hemolytic streptococcus, infection, necrotizing vasculitis, polyarteritis nodosa

## Abstract

**Background:**

Polyarteritis nodosa is a rare systemic necrotizing vasculitis that is often overlooked and misdiagnosed in clinical practice. Patients may exhibit fever, rash, myalgia, and further symptoms; in severe instances, this may result in damage to the kidney, heart, and other important organs, and may even be life-threatening. Consequently, prompt diagnosis and intervention might mitigate the occurrence of complications and improve patient prognosis.

**Patient presentation:**

An 11-year-old girl was admitted to our hospital with multi-joint pain for 7 days, accompanied by worsening fever for 4 days. The physical examination on admission revealed alterations in the skin texture characterized by scaling, a bluish-purple rash, and sensitive subcutaneous nodules on the extremities with limited mobility. Following admission, laboratory testing revealed high serum inflammatory markers, and positive anti-chain “O,” rheumatic fever was initially considered. The symptoms were not relieved after the use of antibiotics and aspirin. After reviewing the literature, polyarteritis nodosa was highly suspected, and a skin biopsy indicated necrotizing vasculitis, therefore confirming polyarteritis nodosa. The child's symptoms were alleviated with the use of glucocorticoids in conjunction with immunosuppressive medication.

**Conclusion:**

This case involves a child diagnosed with nodular polyarteritis subsequent to a streptococcal infection. For patients with a strong suspicion of polyarteritis nodosa, a timely skin biopsy or arterial angiography should be conducted to confirm the diagnosis and increase survival rates.

## Introduction

Polyarteritis nodosa (PAN) is an uncommon necrotizing vasculitis of unclear origin. The disease mostly affects small- and medium-sized arteries in a segmental distribution and can involve multiple organ systems, resulting in complicated and variable clinical symptoms, predominantly characterized by fever, myalgia/arthralgia, rash, and damage to the afflicted organs (1). In Europe, the prevalence of PAN in adults is shown to be 0.002‰–0.009‰ (2), while the incidence in children is considerably lower, posing significant challenges in diagnosis and treatment. This paper reports the clinical data of a case with PAN admitted to Wuhan Children's Hospital. Through a study of pertinent literature, the clinical features of children with PAN were analyzed comprehensively to enhance physicians’ comprehension of the condition and the standards of diagnosis and treatment.

## Patient and observation

### Patient information

A previously healthy 11-year-old girl was admitted to the Rheumatology and Immunology Department of Wuhan Children's Hospital on 16 July 2024 with “multi-joint pain with progressive aggravation for 7 days and increased fever for 4 days.” The patient's parents were healthy and did not have any family diseases. On 10 June 2024, she was diagnosed with acute tonsillitis due to fever and sore throat at the local Maternal and Child Health Hospital. Anti-chain “O” (ASO) and throat swab tests were not performed, and her symptoms improved after antibiotic treatment (the patient's parents did not know the specific antibiotic name). Two days before coming to our hospital, she went to the local hospital and tested positive for ASO but did not receive antibiotic treatment. Upon admission, the patient presented with a range of symptoms, including severe pain in multiple joints, fever exceeding 38℃, and multiple erythematous nodules 1–5 cm in diameter in both lower limbs.

### Clinical findings

**Figure 1:**
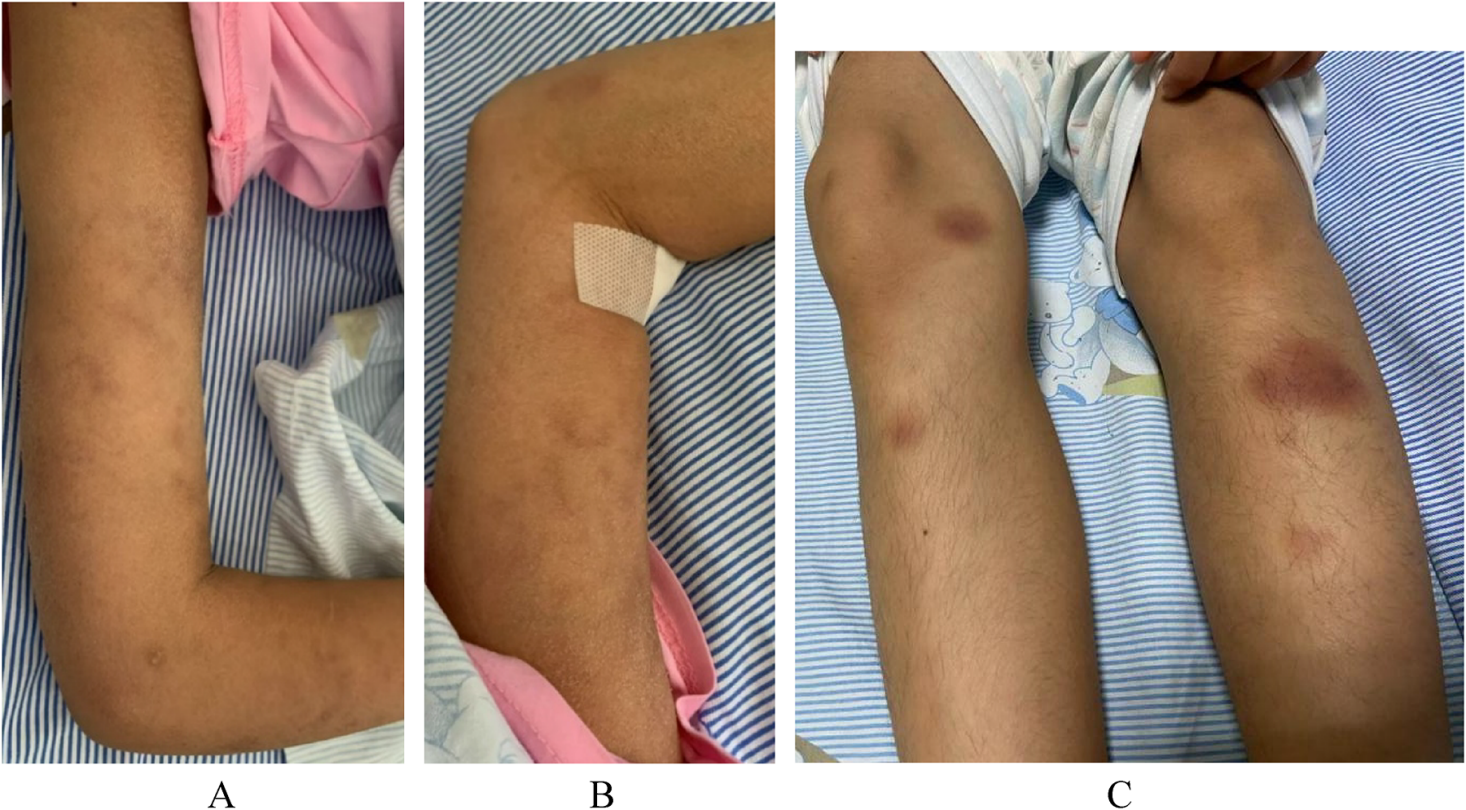
Patient skin changes. **(A)** Livedo reticularis on the right upper limb. **(B)** Livedo reticularis on the left upper limb. **(C)** Purplish-red subcutaneous nodules in both lower limbs.

The physical examination revealed that the skin over her entire body was rough and dry, with a scaly appearance without ulceration, atrophy, or pigmentation changes. There was livedo reticularis on both upper limbs and a purplish-red nodular rash on both lower limbs, accompanied by tenderness. Furthermore, tenderness and swelling of the right shoulder joint; the metacarpophalangeal and wrist joints of the left upper limb; and the phalangeal, ankle, knee, and hip joints of both lower limbs were present, accompanied by mobility disorders. No significant abnormalities were detected in the cardiac, abdominal, and neurological physical examinations (Figure 1).

### Diagnostic assessment

**Figure 2:**
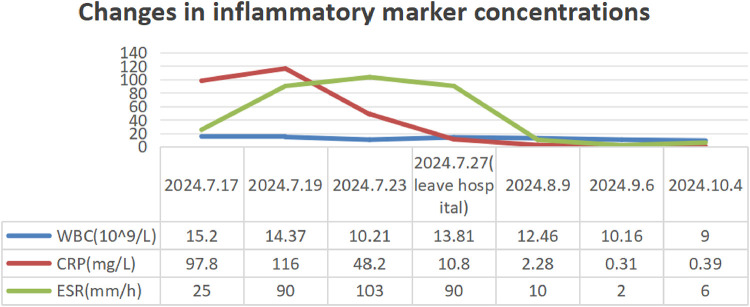
Changes in inflammatory marker concentrations in the patient from July to October 2024.

**Figure 3:**
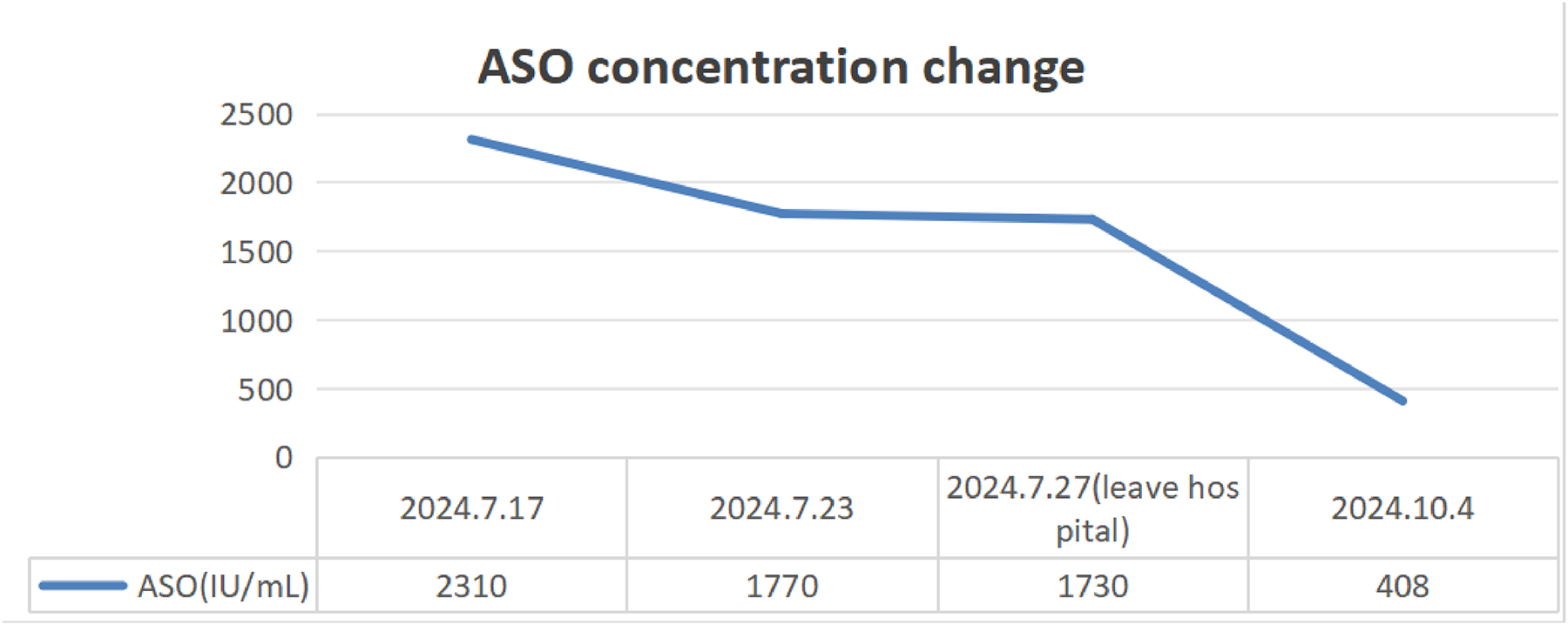
ASO concentration change in the patient from July to October 2024.

**Figure 4:**
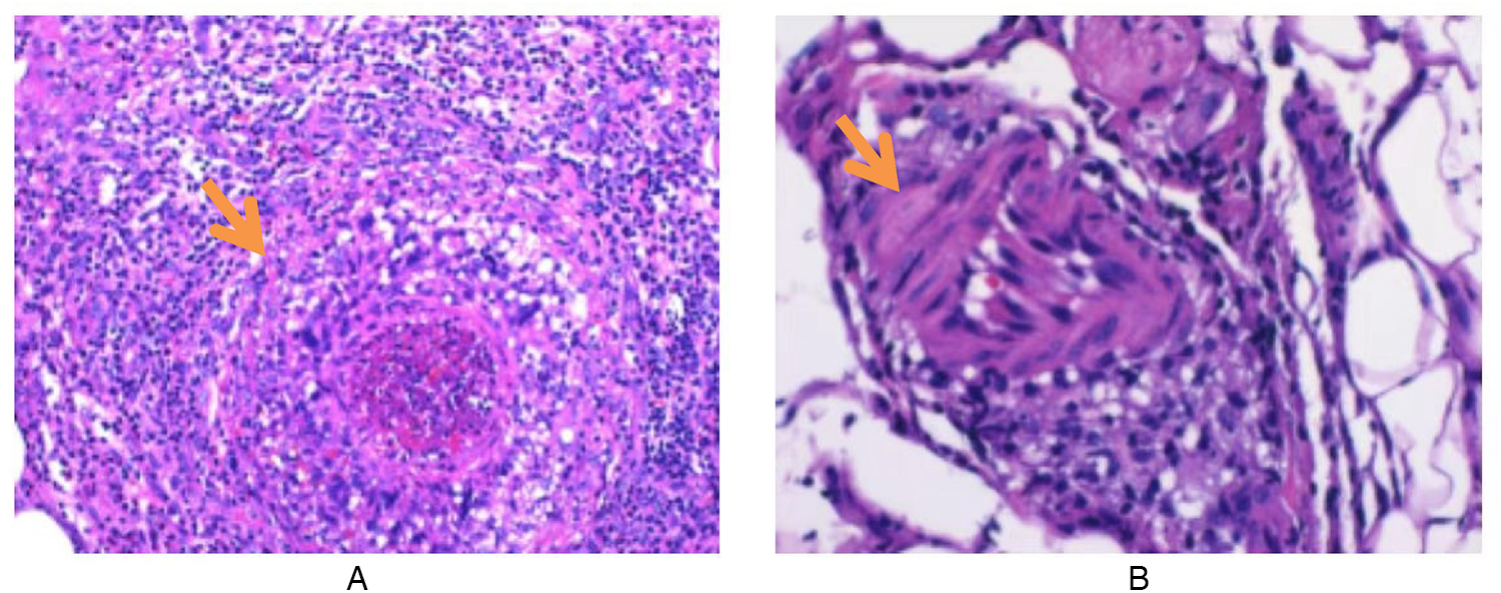
Skin histopathological changes in a child with polyarteritis nodosa. **(A)** Infiltration of inflammatory cells around subcutaneous blood vessels. **(B)** Adipose blood vessel wall degeneration, necrosis, nuclear debris formation, and perivascular inflammatory cell infiltration (hematoxylin–eosin stain; original magnification ×200).

Routine laboratory testing indicated increased serum levels of the following inflammatory markers (Figure 2): white blood cells at 15.20 × 10^9^/L (normal: 4.3–11.3 × 10^9^/L), C-reactive protein (CRP) at 97.8 mg/L (normal: 0–3 mg/L), erythrocyte sedimentation rate (ESR) at 90 mm/h (normal: 0–20 mm/h), ferritin at 215.01 ng/ml (normal range: 12–135 ng/ml), and procalcitonin at 0.15 ng/ml (normal: <0.05 ng/ml). The concentrations of ASO over time (Figure 3) were 2,310 IU/ml (2024.7.17), 1,770 IU/ml (2024.7.23), and 1,730 IU/ml (2024.7.27) (normal: 0–408 IU/ml). The blood cultures showed negative results. Serological tests for HIV, hepatitis B and C antibodies, cytomegalovirus, Epstein–Barr virus, and parvovirus B19 were negative. The tuberculin skin test was negative. All parasites tested negative. The immunological evaluation did not detect antinuclear antibodies, rheumatoid factor, human leukocyte antigen B27 (HLA-B27), anticardiolipin antibody, anti-β2-glycoprotein 1 immunoglobulin G (IgG), anti-β2-glycoprotein 1 immunoglobulin M (IgM), or anti-neutrophil cytoplasmic antibodies. Bone marrow aspiration excluded hematological disorders. No aneurysms or stenosis were detected with color ultrasonography of the limbs. A skin biopsy was completed (Figure 4) and the epidermis exhibited atrophy and flattening, collagen fiber hyperplasia was observed in the dermis, and lymphocyte infiltration surrounding the dermal and subcutaneous veins was identified. Vascular wall degradation, necrosis, and nuclear debris production in subcutaneous adipocytes were observed, and histiocytes, lymphocytes, and granulocytes exhibited vascular-centered infiltration, which was consistent with the signs of vasculitis.

### Therapeutic interventions

The patient had a history of prior infections and presented with recurrent fever, multiple joint swelling and discomfort, and subcutaneous nodular soreness in the limbs. Rheumatic fever was initially considered for diagnosis, and gamma globulin (40 mg/kg/d) was administered for 2 days to suppress inflammation and improve immunity, along with amoxicillin clavulanate potassium (80 mg/kg/day) and aspirin. However, the patient’s body temperature remained high, the joint pain of the limbs was severe without significant improvement, and the subcutaneous nodules of the lower extremities did not subside and were accompanied by tenderness. After the histological manifestations of necrotizing vasculitis were confirmed by the skin biopsy, the patient was treated with methylprednisolone (2 mg/kg/day) for 3 days. In consideration of the long-term efficacy and side effects, mycophenolate was added. Concurrently, aspirin was substituted with dipyridamole as the oral antiplatelet agent for the prevention of thrombosis. The patient’s body temperature gradually normalized, the rash diminished, joint discomfort was mostly alleviated, and the subcutaneous nodules greatly reduced, allowing for patient release and follow-up. Following discharge, prednisone, mycophenolate mofetil, and dipyridamole were maintained, with an oral calcium supplement. The patient and her family were instructed to return to the hospital periodically for the evaluation of relevant indicators and medication guidance. During the follow-up, the patient began treatment with benzathine penicillin and we found that the inflammatory markers of the patient gradually decreased after discharge and ASO gradually returned to normal (Figures 2, 3).

## Discussion

PAN is a rare systemic necrotizing vasculitis that occurs 2.5–4.0 times more frequently in male individuals than in female individuals and primarily affects those aged 40–60 years. The incidence of PAN in adults in Europe has been reported to be 0.002‰–0.009‰ (2), and it is considerably more uncommon in children. The etiology and pathogenesis of PAN remain unidentified and are potentially linked to various infections (including group A hemolytic streptococcus, hepatitis B virus, hepatitis C virus, recurrent urinary tract infections, parvovirus B19, and mycobacterium tuberculosis), autoimmune diseases, and pharmaceuticals. The immunopathological process predominates in the illness. The predominant etiology of cutaneous PAN (CPAN) is β-hemolytic streptococcal infection of group A, with reports indicating that over 80% of cases are associated with streptococcal infection (3), often resulting in the formation of nodules in the lower limbs. Deficiency of ADA2 (DADA2) is a rare genetic disorder caused by a biallelic mutation of the ADA2 gene. Nodular polyarteritis-like symptoms, immune deficiencies, and blood system abnormalities (anemia and thrombocytopenia) are the clinical features of DADA2 (4). Both DADA2 and PAN cause necrotizing vasculitis and severe inflammation. Systemic PAN is a differential diagnosis of DADA2. However, the treatment of the two diseases is very different, as anti-TNF therapy is the first choice for DADA2 disease (5), while PAN therapy is commonly used in combination with corticosteroids and immunosuppressants. Therefore, for children with PAN-like phenotypes, DADA2 should be considered, and screening should include ADA2 level measurement and basal immune deficiency testing to rule out potential organ damage (6). The patient’s ADA2 level was not measured in this case, nor was genetic testing conducted, which is also worthy of our attention. In this case, the patient’s ASO concentration was initially positive and progressively increased along the disease's progression. Based on the clinical manifestations, indicators, and skin biopsy evidence, it was determined that the child had developed PAN following a streptococcal infection, which aligns with existing studies in the literature (3).

PAN mainly affects small and medium muscular arteries, with damage being segmental, frequently occurring at arterial bifurcations, and spreading to distal regions (1). PAN vasculoinflammatory infiltrates are often heterogeneously abundant in neutrophils, lymphocytes, and macrophages, leading to fibrosis, thrombosis, or aneurysmal degeneration. The first vascular pathological alterations of PAN exhibit fibrinoid necrosis, mainly involving neutrophils. Lymphocyte and macrophage infiltration, together with new angiogenesis, are typically observed in advanced vascular disease. Advanced vascular disease presents with intimal hyperplasia and fibrosis. Inflammatory alterations at various phases of vascular circulation are characteristic hallmarks of PAN (7). A skin biopsy confirmed the presence of PAN vasculitis in this case.

PAN can be divided into systemic PAN (SPAN) and CPAN according to whether internal organs are involved. In addition to systemic symptoms such as fever, fatigue, myalgia or arthralgia, and weight loss, SPAN often involves the kidneys, nervous system, and digestive system, leading to hypertension, proteinuria, convulsions, disturbance of consciousness, gastrointestinal bleeding, obstruction, and other related manifestations, among which the heart, lungs, and central nervous system are rarely involved (1, 8). CPAN is limited to the skin without visceral involvement, and may also present with extra-cutaneous manifestations such as fever, weakness, myalgia/joint pain, and peripheral neuropathy. In 2005, the International Pediatric Association proposed a novel EULAR/PRINTO/PRES classification standard for pediatric vasculitis at the Vienna conference (9), which included a new diagnostic criterion for SPAN in children, which is significantly different from the adult criterion. The necessary bases include histopathological evidence of necrotizing vasculitis or angiographic anomalies (aneurysm, stenosis, or occlusion) affecting small or medium arteries, which is accompanied by one of five symptoms: skin involvement, myalgia or muscle tenderness, hypertension, peripheral neuropathy, and renal involvement. Although there is no strict diagnostic definition of CPAN, it is generally considered to be a distinct form of necrotizing vasculitis. In addition to the usual symptoms of fever and fatigue, the patient showed reticular green spots, purplish-red painful subcutaneous nodules, swelling and pain in multiple joints, no signs of kidney or other vital organ damage, and a skin biopsy confirmed the presence of PAN vasculitis, a key component in the diagnosis of CPAN in this child. PAN is often associated with elevated laboratory test values for ESR, CRP, and other acute phase reactants but these lack specificity. This suggests that angiography and histopathology are essential for the diagnosis of PAN.

We conducted a literature review and found a total of six articles consistent with the diagnosis of PAN with streptococcal infection in children under 18 years of age (10–15). Together with this case, we summarized the clinical features and prognosis of 19 cases of PAN associated with streptococcal infection in children (Table 1). The male:female ratio was 2.8:1. The median age was 8 years (range 2–13 years). All 19 patients (100%) had skin lesions, 18 patients (94.7%) had a positive ASO test or positive throat swab, 17 patients (89.5%) had high fever, 16 patients (84.2%) had joint symptoms, and 2 patients (10.5%) had myalgia, none of which involved organs. In five patients (26.3%), rashes and joint symptoms recurred, all of which were accompanied by streptococcal infection symptoms such as fever and pharyngeal pain, and a positive ASO/throat swab. All 19 patients had histopathological evidence from a skin biopsy. Through a systematic review of the literature, we found that the incidence of streptococcal infection-associated PAN is low in children, and the recurrence of the disease is related to repeated streptococcal infection. The relevant mechanism needs to be further studied. High fever, skin lesions, and joint symptoms are present in almost all patients, organ involvement is rare, and the prognosis is good.

**Table 1:** The clinical features and prognosis of PAN associated with streptococcal infection in children since 2005 (*N* = 19).

Case	Total *(N)*	Sex	Age (years)	Fever	Skin involvement (subcutaneous nodules, livedo reticularis, purpura, edema, and/or ulcers)	Joint involvement (arthralgia, arthritis)	Muscle involvement (myalgias, myositis)	Affected organs (nervous system, kidneys, digestive system, etc.)	ASO (+)/oropharyngeal swab (+)	Follow-up (recurrence/death)	Reference
Case 1	1	M	8	1 (100%)	1 (100%)	1 (100%)	—	—	1 (100%)	—	(10)
Case 2	10	8M 2F	7–13	8 (80%)	14 (100%)	9 (90%)	1 (10%)	—	10 (100%)	Relapse in three cases	(11)
Case 3	1	F	4	1 (100%)	1 (100%)	—	—	—	1 (100%)	Recurrence	(12)
Case 4	1	M	8	1 (100%)	1 (100%)	1 (100%)	1 (100%)	—	1 (100%)	Recurrence	(13)
Case 5	1	M	6	1 (100%)	1 (100%)	1 (100%)	—	—	1 (100%)	—	(14)
Case 6	4	3M 1F	2–10	4 (100%)	4 (100%)	3 (75%)	—	—	3 (75%)	—	(15)
Ours	1	F	11	1 (100%)	1 (100%)	1 (100%)	—	—	1 (100%)	—	—

The location of affected organs and the development of the illness are the primary factors that determine treatment in individuals with PAN. The current therapy for PAN mainly involves glucocorticoids combined with immunosuppressants. In cases of life-threatening conditions or rapid disease development, methylprednisolone impact treatment (1 g/day, 3–5 days) may be administered. In cases with cutaneous PAN without organ involvement, prednisone can be taken orally at a dosage of 1 mg/kg/day, with slow tapering following remission. When prednisone cannot be tapered below 15–20 mg/day, when patients with PAN are intolerant to the adverse effects of prolonged hormonal therapy, or when critical organs are implicated, immunosuppressants may be considered, with cyclophosphamide being the preferred option. Methotrexate, azathioprine, or mycophenolate may also be utilized. Methotrexate acts more rapidly. Nonetheless, it should be contraindicated in those with renal and hepatic disorders. Moreover, the anticoagulants aspirin and dipyridamole may also be utilized. Complications, including gastrointestinal tract perforation/rupture or renal hemorrhage, may necessitate surgical intervention. In the presence of streptococcal infection or hepatitis B virus infection, penicillin anti-infective medication or antiviral therapy is crucial for effective treatment and recurrence prevention (7, 8, 11). Despite the absence of significant organ injury in the child, the involvement of multiple limb joints and restricted mobility necessitated the administration of prednisone, mycophenolic acid, the anticoagulant dipyridamole, and penicillin to fight the infection, resulting in substantial improvement in the child's clinical symptoms post-treatment.

Untreated patients with PAN have a poor prognosis, with a 5-year survival rate of approximately 13%, which improves to as high as 80% following conventional therapy (16). PAN, while rare in clinical practice, can be life-threatening if it affects vital organs. Consequently, it is suggested that pediatricians remain vigilant for the potential occurrence of PAN in clinical practice when faced with an unexplained fever accompanied by myalgia, arthralgia, and subcutaneous nodules, in addition to ruling out neoplastic illnesses and rheumatic fever. For children, if arterial angiography cannot be conducted, a skin biopsy should be promptly performed to provide early diagnosis and treatment, thereby improving the prognosis. However, it is important to acknowledge that there are several limitations in our case report. First, genetic testing for DADA2 was not performed, which could have provided more informed information about potential differential diagnoses. Another limitation is that oral and pharyngeal swabs were not collected from the patient and only ASO concentration was tested multiple times. Despite these limitations, this study provides clinicians with a broader understanding of PAN manifestations and may have implications for the future diagnosis and management of PAN.

## Conclusion

This case report highlights the importance of maintaining a high degree of suspicion for rare diseases such as PAN in children, and the strong association observed between PAN and streptococcal infections should also be noted. Therefore, it is necessary to study the etiology and pathogenesis of PAN, which can help clinicians understand and manage this disease more effectively.

## Data Availability

The original contributions presented in the study are included in the article/Supplementary Material, further inquiries can be directed to the corresponding authors.
